# High fever with rash: a case report of spotted fever group rickettsial infection at a construction site

**DOI:** 10.1186/s12879-026-12735-7

**Published:** 2026-02-02

**Authors:** Luhang Li, Jiahui Wang, Zekun Li, Dong Xing

**Affiliations:** The Fourth Hospital of Hebei Medical University, Shijiazhuang City, Hebei Province China

**Keywords:** Construction worker, Sanitary environment, Rickettsial infection, High fever, Rash

## Abstract

**Background:**

Spotted Fever Group Rickettsiae (SFGR) infection is one of the global public health threats. With the improvement of current hygiene conditions, the incidence of rickettsial infections has significantly decreased compared with previous years; however, in clinical practice, rickettsial infections should still be considered in the differential diagnosis of febrile cases of unknown etiology.

**Case presentation:**

A construction worker, residing in crowded and poor sanitary conditions, presented with high fever and diffuse cutaneous rash, without reporting other associated discomfort. Empirical treatment with cephalosporin antibiotics failed to alleviate the symptoms. Given the unknown etiology of the patient’s high fever and rash, routine etiological tests yielded negative results, with concurrent abnormalities in the white blood cell differential count of the complete blood count. A strong suspicion of infection by an unusual microorganism prompted the performance of metagenomic next-generation sequencing (mNGS) on venous blood. This test identified infection with Rickettsia rickettsii belonging to the spotted fever group, confirming a rickettsial infection. Following the establishment of the etiology, the antimicrobial treatment regimen was adjusted, and the patient was administered doxycycline for antimicrobial therapy. After treatment, the patient’s body temperature returned to normal, the rash resolved, and the patient was discharged in a state of full recovery.

**Summary:**

For patients working at construction sites with poor living conditions who present with high fever and rash but lack evidence of conventional microbial infection, clinicians should enhance their differential diagnostic capabilities and maintain vigilance for the occurrence of rickettsial infection.

**Supplementary Information:**

The online version contains supplementary material available at 10.1186/s12879-026-12735-7.

## Background

The epidemiological history of Spotted Fever Group Rickettsiae (SFGR) can be traced back to 1906, when Rickettsia rickettsii was first detected in the United States, where it causes Rocky Mountain spotted fever. Since that time, SFGR has gradually been acknowledged as a global public health hazard. According to literature reports, between 1906 and 2021, 48 SFGR species have been documented worldwide, involving 66,133 human infection cases, with substantial variations in their spatial distribution. The Americas, the Mediterranean region, and East Asia are the primary endemic areas for SFGR infections. Ticks, serving as the principal vectors for SFGR, have undergone shifts in their ecological niche—such as expansions in their distribution range and overlaps between their activity periods and human outdoor activity cycles—and these changes have further contributed to the spread of these pathogens [1]. In China, Japanese spotted fever has emerged as one of the most prevalent rickettsial diseases in comparison to other types of rickettsial infections. Climate and environmental changes may exert a significant impact on the epidemiology of Japanese spotted fever. This article describes the diagnosis and treatment of a cluster of spotted fever group rickettsial infection cases among construction workers living in crowded, substandard housing. It aims to remind clinicians to recognize the high-risk factors for such infections in this specific population.

## Case presentation

**Fig. 1:**
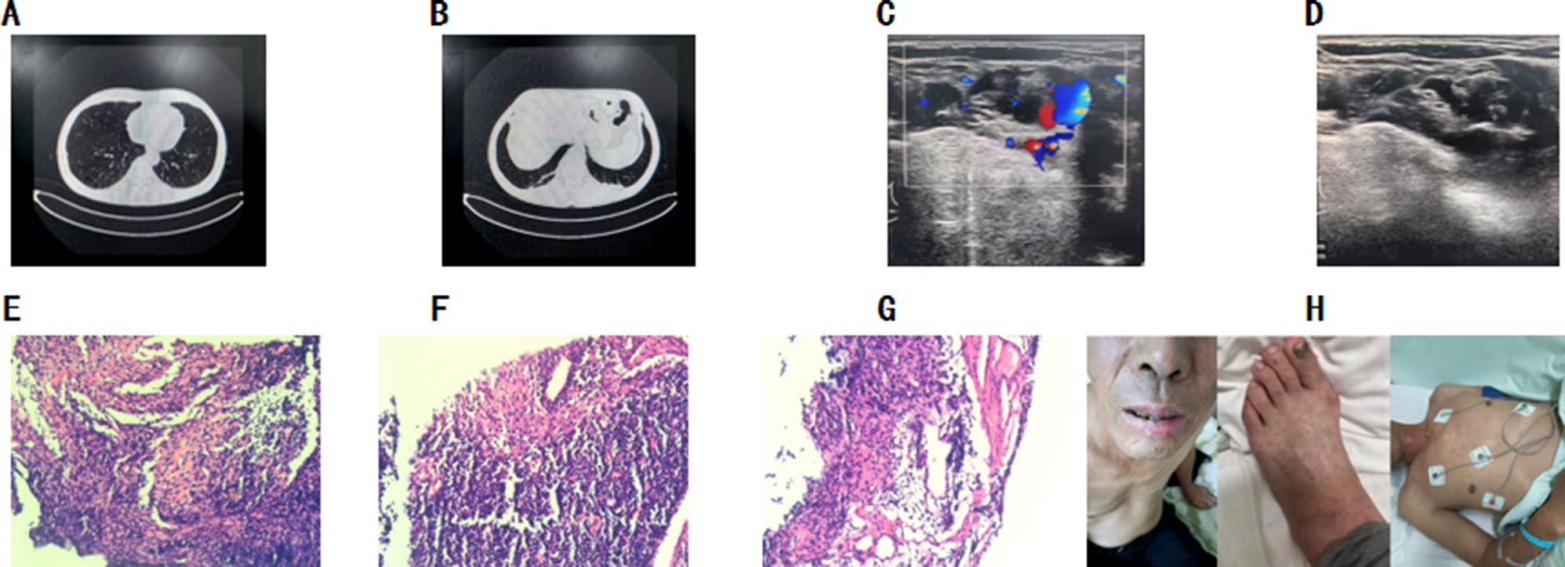
Relevant examination results. (**A**, **B**) Head, chest, and abdominal CT findings; (**C**–**G**) Neck lymph node ultrasound and pathological biopsy results; (**H**) The appearance of suspicious oral plaques during the patient’s treatment

The patient was a 57-year-old male construction worker, admitted to the hospital with the chief complaint of “high fever accompanied by generalized rash for 5 days.” He resided in a collective housing facility at a construction site with poor living conditions. Five days prior to admission, he developed generalized fatigue, which did not improve with rest. He self-measured his body temperature at 38.3 °C (100.9°F) and received cephalosporin antibiotics at a community hospital. However, his fever did not improve significantly and peaked at 40.8 °C (105.4°F), prompting him to seek medical attention at our emergency department. On admission, his body temperature was 38.6 °C (101.48°F), and scattered rashes were observed on the trunk and extremities; these rashes did not blanch upon pressure. Head, chest, and abdominal CT findings: A small solid nodular shadow in the right lower lobe near the oblique fissure; A pulmonary bulla in the right lower lobe; Minimal linear patterns in both lower lobes; Localized thickening of the lower dorsal pleura bilaterally. Focal lacunar infarction in the head of the left caudate nucleus; No other abnormalities noted on plain cranial scanning. No significant abnormalities identified on plain abdominal scanning (Fig. 1A, B). Laboratory test results: Rapid C-reactive protein (149.56 mg/L) and neutrophil percentage (94.2%) were significantly elevated, while lymphocyte percentage (4.2%) and platelet count (62 × 10^9^/L) were markedly decreased. The red blood cell count (3.94 × 10^12^/L) was mildly reduced, and the white blood cell count was within the normal range (Table 1). Urinalysis, novel coronavirus nucleic acid testing, 13-pathogen respiratory panel, hepatitis A/B/C antibodies, HIV antibody, and syphilis serology all yielded negative results (Table 1, Fig. S1).

**Table 1:** Relevant laboratory test results

Item	Reference Range	Date					
		Prior to admission	At admission				
		5–6	5–6	5–7	5–9	5–11	5–14
2019 novel coronavirus	Negative		Negative				
13 kinds of pathogens	Negative		Negative				
CRP (mg/L)	0–4	149.56					
Routine blood test							
WBC (*10^9^/L)	3.5–9.5	5.19	4.54	3.7	7.34	9.18	6.59
NE (%)	40–75	94.2	94.4	86.5	83.2	60.4	52.8
LY (%)	20–50	4.2	3.2	12.8	12.7	34.6	38
MO (%)	45726	1.6	2.4	0.7	4.0	3.8	7.3
RBC (*10^12^/L)	4.3–5.8	3.94	3.61	3.55	3.57	3.82	3.58
HGB (g/L)	130–175	132.0	120.0	118	117	126	117
PLT (*10^9^/L)	125–350	62	60	67	102	194	245
Coagulation							
PT (s)	9.4–12.5	11.4		10.4	11.1	10.9	10.4
APTT (s)	25.1–36.5	40.1		33.3	30.1	28.5	29.4
D-DIMER (mg/L)	＜0.243	1.053		0.804	0.287	0.153	0.209
FIB (g/L)	2.38–4.98	4.18		4.21	3.74	4.71	4.13
Biochemistry Test							
Na (mmol/L)	137–147		129.0	129.0	132.0	136.0	139.0
Ca (mmol/L)	2.11–2.52			2.02	2.08	2.27	2.17
ALT (U/L)	9–50			78.0	56.6	49.8	40.3
AST (U/L)	15–40			38.7	31.4	28.7	28.9
ALB (g/L)	40–55			29.7	29.1	33.4	32.1
Cr (umol/L)	57–97		90.0	82.0	74.0	77.0	73.0
UREA (mmol/L)	3.1–8		5.6	5.7	4.1	4.7	4.2
BDG (pg/ml)	Negative: ＜100.5					29.1	
	Positive: ≥100.5						
GAL(S/CO)	Negative: ＜0.5					0.14	
	Positive: ≥0.5						

Notes: Abbreviations: HIV Human Immunodeficiency Virus; TP: Treponema pallidum; CRP C-reactive protein; WBC White blood cell count; NE Neutrophil; LY Lymphocyte; MO Monocyte; RBC Red Blood Cell; HGB Hemoglobin; PLT Platelets count; PT Prothrombin time; APTT Activated partial thromboplastin time; FIB Fibrinogen; Na Sodium Ion Calcium; Ca Calcium Ion; ALT Alanine transaminase; AST Aspartate transaminase; ALB Albumin; Cr Creatinine; UREA Urea; BDG (1,3)-β-D-glucan; GAL Aspergillus galactomannan

**Fig. 2:**
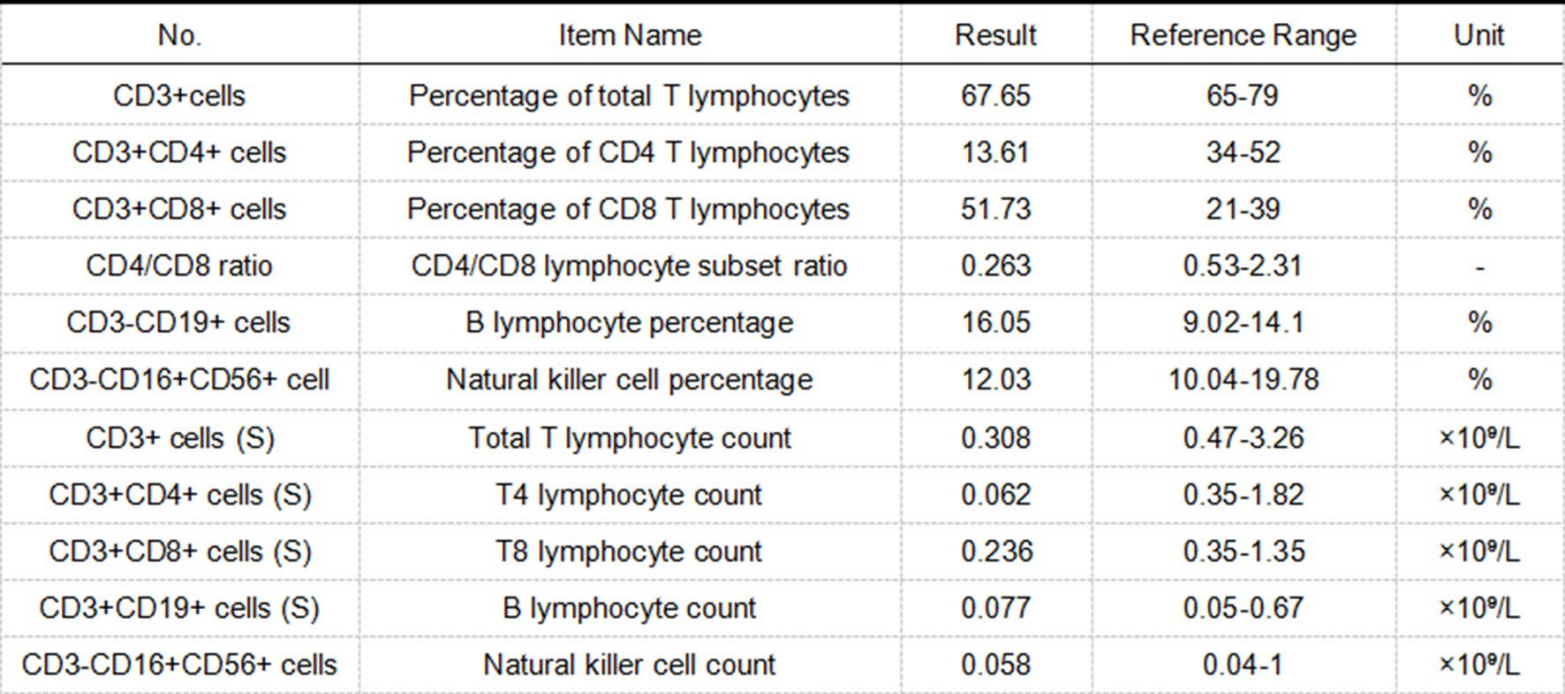
Peripheral blood lymphocyte subset testing

**Fig. 3:**
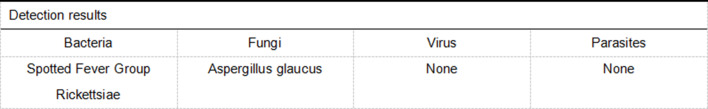
Results of mNGS

Upon admission, the patient was empirically treated with piperacillin-tazobactam for suspected infection. Prior to the administration of antimicrobial agents, blood samples (for aerobic and anaerobic cultures) were collected for microbiological testing, which returned negative results. Testing for fungal markers (including (1,3)-β-D-glucan [BDG] and Aspergillus galactomannan [GAL]) (Table 1) and peripheral blood lymphocyte subset analysis (Fig. 2) were completed. Neck lymph node ultrasound and pathological biopsy results (Fig. 1C–G): Multiple lymph nodes in the right cervical IV region showed cortical thickening, with the largest measuring approximately 1.8 × 1.1 cm. Multiple lymph nodes in the right supraclavicular region also exhibited cortical thickening, with the largest measuring approximately 1.0 × 0.9 cm. Histopathological examination of the lymphoid tissue obtained via puncture revealed histiocytic infiltration. Given the patient’s lack of response to antimicrobial therapy at the community hospital, the possibility of infection by an unusual microorganism could not be ruled out. Venous blood metagenomic next-generation sequencing (mNGS) was therefore performed. Laboratory results showed that the detection value of fungal (1,3)-β-D-glucan (BDG) was 29.1 pg/ml, and the detection value of Aspergillus galactomannan (GAL) was 0.14 S/CO, both of which were negative. The mNGS results (Fig. 3) indicated infections with Rickettsia rickettsii (8 sequences, 0.12% relative abundance) and Aspergillus glaucus (16 sequences, 42.1% relative abundance) in the patient’s blood. The diagnosis was confirmed as Rickettsia rickettsii infection. The antimicrobial regimen was adjusted to oral doxycycline (200 mg on day 1, followed by 100 mg once daily) combined with voriconazole (400 mg every 12 hours on day 1, followed by a maintenance dose of 200 mg every 12 hours) for a one-week period. On day 3 of treatment, the patient’s body temperature returned to normal, the rash gradually subsided, and infection-related indicators progressively returned to the normal range (Fig. S2). The patient ultimately achieved a full recovery and was discharged from the hospital.

## Discussion and summary

In China, spotted fever group rickettsioses (SFGR) are predominantly caused by Rickettsia japonica infection, and this infectious disease is transmitted by ticks. Since its initial detection in Hainan Province in 1989, the epidemic scope and the number of infections have expanded significantly. Currently, human cases have been reported in 14 provinces, and tick vectors have been detected in 19 provinces. Research indicates that the endemic area of Japanese spotted fever (JSF) has gradually expanded from the eastern coastal regions to the western inland areas, with the humid mountainous regions of Central and Eastern China forming the core high-incidence zones. Rural residents aged 55 years and above, who engage in long-term farming and outdoor work, constitute the most prominent high-risk group, accounting for over 70% of all cases due to frequent tick exposure [2]. In recent years, multiple reports of rickettsial infections have emerged in North China. While direct case reports from construction sites are scarce, CDC surveillance data indicate high tick densities in the vicinity of construction sites. Given the large and concentrated population of construction workers in China, this situation suggests the need for further epidemiological investigations to clarify the infection status at construction sites. This highlights that poor sanitation remains a significant contributing factor to rickettsial infections.

In this case, the patient was a construction worker exposed to poor living and working conditions. The current illness primarily presented with high fever and generalized rash, accompanied by abnormal laboratory findings, including a complete blood count showing a white blood cell count of 4.54 × 10^9^/L, a neutrophil percentage of 94.4%, a lymphocyte percentage of 3.2%, and a platelet count of 60 × 10^9^/L, as well as elevated C-reactive protein (149.56 mg/L) and procalcitonin (2.5 ng/mL). Based on the patient’s occupational history and the negative pre-admission results for HIV antibody, syphilis serology, novel coronavirus nucleic acid, and 13-pathogen respiratory panel, a viral infection was deemed unlikely, suggesting a higher probability of infection by an unusual microorganism. The significant reduction in lymphocyte percentage in the patient’s complete blood count prompted performance of peripheral blood lymphocyte subset testing (Fig. 2). Results revealed the patient is currently in a state of immune dysregulation, with an elevated risk of opportunistic infections. Moreover, as the disease progresses, the likelihood of concurrent multiple opportunistic infections rises significantly. Given the lengthy duration and limited accuracy of conventional blood culture testing (which ultimately yielded negative results), blood mNGS testing was initiated immediately to rapidly control the patient’s condition. The results indicated infections with Rickettsia and Aspergillus glaucus. Notably, Rickettsia is an intracellular parasite, which is consistent with the preliminary diagnosis. Since Rickettsia cannot grow on conventional culture media, the negative blood culture results in this case did not rule out Rickettsia infection [3], highlighting the limitations of traditional culture methods for diagnosing such pathogens.

Laboratory-assisted diagnostic methods for rickettsial infections are diverse, with varying sensitivities and specificities in test results. Currently, seroepidemiological studies targeting spotted fever group rickettsiae (SFGR) in Asia predominantly employ IFA (indirect immunofluorescence assay) and ELISA (enzyme-linked immunosorbent assay). IFA is considered the “gold standard” for quantitative detection of rickettsial antibodies, particularly effective in distinguishing different rickettsial diseases [4]. However, in cases where the etiology remains unclear, blood mNGS testing plays a crucial role. mNGS testing offers high sensitivity and specificity, enabling the unbiased detection of all microbial nucleic acids in clinical samples. This significantly enhances pathogen detection rates, particularly for the diagnosis of infections of unknown etiology, and provides critical evidence for the timely adjustment of clinical treatment strategies. In this patient, although blood mNGS detected a relatively low abundance (0.12%) of spotted fever group rickettsiae, it is important to note that rickettsiae are obligate intracellular parasites. Once detached from living cells, they cannot replicate and are readily eliminated by the host immune system and antibiotics. Therefore, detecting live rickettsial DNA in routine blood cultures or traditional PCR tests is extremely difficult. When peripheral blood mNGS captures its sequence—even at a relative abundance of just 0.12%—it indicates that the actual load within target cells far exceeds 0.12%. This discrepancy arises because the parasites reside intracellularly and are not fully captured by sequencing. Thus, the detection of Rickettsia in blood itself indicates substantial intracellular colonization in the patient’s cells [5]. In this patient, environmental factors played a significant role in the occurrence of infection. Through mNGS technology, we detected two microorganisms: Rickettsia and Aspergillus glaucus. Combining the patient’s work environment, clinical manifestations, and treatment response, SFGR was identified as the definitive pathogen. However, the status of Aspergillus glaucus (infection vs. contamination) remains questionable. The presence of suspected oral plaque during treatment (Fig. 1H) and the blood mNGS results only indirectly suggest a possible fungal infection, with no definitive diagnostic evidence. Transient immunosuppression due to persistent fever and systemic inflammatory stress (a high-risk factor for opportunistic infections) may be one of the reasons for the detection of Aspergillus glaucus in the blood. Therefore, antifungal therapy remains clinically necessary for this patient. Based on the diagnosis and treatment experience of this case, for patients suspected of Rickettsia infection, especially those with skin eschars, priority should be given to collecting eschar or skin swab samples. Specific screening for Rickettsia using molecular biology techniques or mNGS can further improve the targeting and accuracy of etiological diagnosis.

In terms of treatment, doxycycline, as the drug of choice for rickettsial infections, demonstrated excellent efficacy in this case. This case underscores the critical importance of timely and accurate pathogen diagnosis and targeted therapy, which were key factors contributing to the patient’s recovery. Simultaneously, this case highlights the limitations of early empirical antibiotic therapy. Failure to promptly identify the causative agent may lead to treatment delays, increasing both the patient’s treatment costs and the risk of complications.

Rickettsial infections present diverse clinical manifestations, ranging from mild self-limiting courses to fatal outcomes. Due to nonspecific acute febrile symptoms (e.g., fever, chills, headache) and occasional maculopapular rashes or vector-bite eschars [6, 7], they are highly susceptible to misdiagnosis, missed diagnosis, or delayed diagnosis. As noted, Japanese spotted fever (JSF) has expanded endemically from eastern coastal to western inland regions, with increased rickettsial infection incidence in northern areas. Poor sanitation and high arthropod density remain high-risk settings. Clinicians in North China must enhance awareness: for atypical fever-rash cases (especially high-risk groups like crowded construction workers with unexplained fever), detailed history-taking, standardized physical examination, and targeted pathogen detection are essential for prompt etiological diagnosis, targeted treatment, and improved prognosis.

In summary, accelerated globalization, increased population mobility, and significant ecological changes may have substantially altered the epidemiological characteristics of rickettsial infections, resulting in considerable potential transmission risks. Against this backdrop, clinicians must continuously enhance their understanding of rickettsial infections while actively promoting the application of specialized pathogen detection technologies. This approach will enable more effective management of such infectious diseases, thereby safeguarding patient health and lives.

## Electronic supplementary material

Below is the link to the electronic supplementary material.

Supplementary Material 1

## Data Availability

Data is provided within the manuscript or supplementary information files.

## Abbreviations

SFGR: Spotted Fever Group Rickettsiae
CT: Computed tomography
mNGS: Metagenomic next-generation sequencing
JSF: Japanese spotted fever
IFA: Indirect immunofluorescence assay
ELISA: Enzyme-linked immunosorbent assay
PCR: Polymerase Chain Reaction
