# Supplementary material for: High fever with rash: a case report of spotted fever group rickettsial infection at a construction site
Source: BMC Infect Dis. 2026 Feb 2;26:472. doi: 10.1186/s12879-026-12735-7 (PMC12952137; doi:10.1186/s12879-026-12735-7)

**Fig. S1** Results of COVID-19 testing and 13-pathogen panel detection


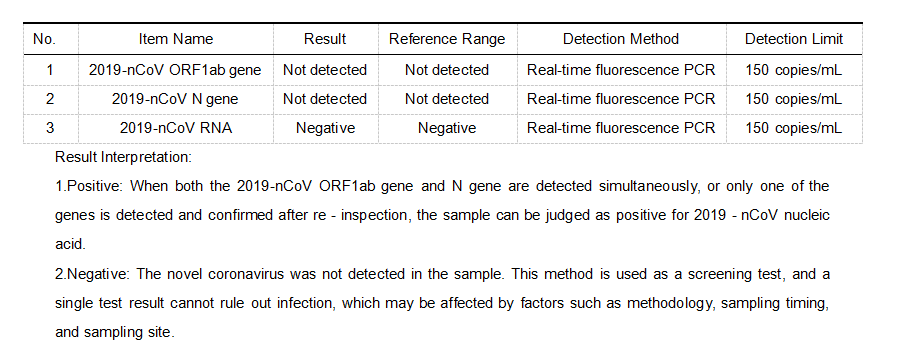


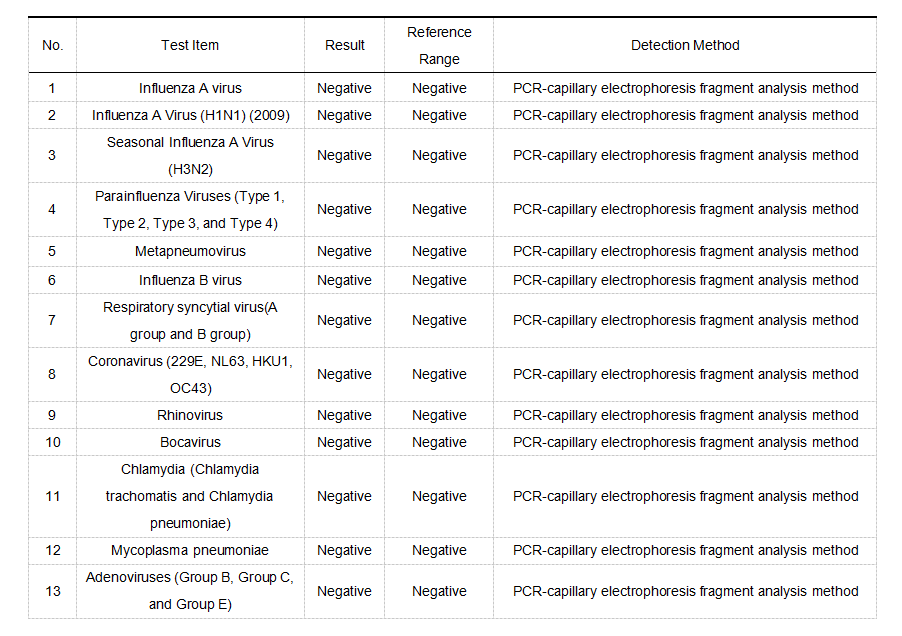


**Fig. S2** Dynamic Changes of Peripheral Blood WBC and Its Subpopulations


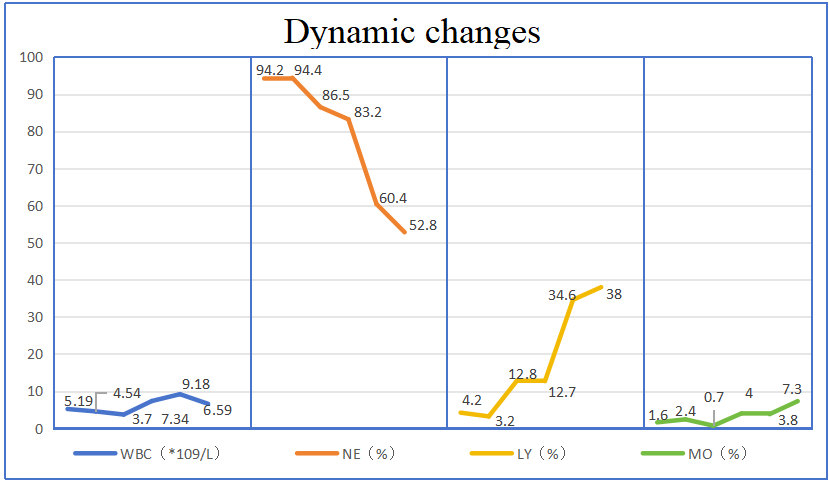

Supplement: Supplementary file 1 — Supplementary Material 1 [file 12879_2026_12735_MOESM1_ESM.docx]
